# Atrophy of amygdala and abnormal memory-related alpha oscillations over posterior cingulate predict conversion to Alzheimer’s disease

**DOI:** 10.1038/srep31859

**Published:** 2016-08-22

**Authors:** Laura Prieto del Val, Jose L. Cantero, Mercedes Atienza

**Affiliations:** 1 Laboratory of Functional Neuroscience, CIBERNED, Network Center for Biomedical Research in Neurodegenerative Diseases, Pablo de Olavide University, Seville, Spain

## Abstract

Synaptic dysfunction, a key pathophysiological hallmark of Alzheimer’s disease (AD), may account for abnormal memory-related EEG patterns in prodromal AD. Here, we investigate to what extent oscillatory EEG changes during memory encoding and/or retrieval enhance the accuracy of medial temporal lobe (MTL) atrophy in predicting conversion from amnestic mild cognitive impairment (aMCI) to AD. As expected, aMCI individuals that, within a 2-year follow-up period, developed dementia (N = 16) compared to healthy older (HO) (N = 26) and stable aMCI (N = 18) showed poorer associative memory, greater MTL atrophy, and lower capacity to recruit alpha oscillatory cortical networks. Interestingly, encoding-induced abnormal alpha desynchronized activity over the posterior cingulate cortex (PCC) at baseline showed significantly higher accuracy in predicting AD than the magnitude of amygdala atrophy. Nevertheless, the best accuracy was obtained when the two markers were fitted into the model (sensitivity = 78%, specificity = 82%). These results support the idea that synaptic integrity/function in the PCC is affected during prodromal AD and has the potential of improving early detection when combined with MRI biomarkers.

AD has now reached the loaded position of being recognized as a public worldwide health priority. In the absence of an effective therapy, reliable approaches to early diagnosis provide the best opportunity for future disease-modifying treatments to prevent and/or delay irreversible pathological changes^1^,^2^. Consistent with this view, new research criteria for preclinical AD have included biomarkers of neurodegeneration and amyloid-β (Aβ) deposition. Converging evidence suggests that they are quite heterogeneous in predicting progression to AD, supporting the idea that different biomarkers become abnormal at different stages of the disease^3^.

Even though the two major proteinopathies underlying AD, Aβ accumulation and neurofibrillary tangles, may be initiated independently^3^, they both cause neuronal and synapse loss in the very early stages of the disease^4^,^5^, leading to progressive dysfunction of neural networks controlling memory processes. Accordingly, functional magnetic resonance imaging (fMRI) studies have provided correlates of heightened Aβ burden in clinically normal adults associated with abnormal patterns of resting-state functional connectivity^6^,^7^,^8^ and with functional deficits in brain areas subserving memory^9^,^10^,^11^. This evidence has recently been extended to resting-state cortical networks in MCI^12^,^13^.

Whereas fMRI has the capacity to measure hemodynamic responses associated with neuronal activity, EEG/MEG recordings provide a more direct measure of synaptic activity in cortical regions. It is, therefore, no wonder that EEG oscillations in a working-memory task^14^ and a resting-state condition with closed eyes^15^ were highly accurate in distinguishing at baseline stable MCI from those that developed AD one year later. However, to the best of our knowledge, no study to date has evaluated whether abnormal EEG oscillations associated with episodic/semantic memory impairment are also able to predict the risk of conversion from MCI to AD.

We hypothesize that power modulations of posterior EEG alpha sources associated with memory performance would be a good predictor of dementia in aMCI, especially if they are combined with MRI biomarkers of AD. Various lines of research support this hypothesis. Firstly, results derived from computational models have shown that cortical and thalamic oscillations in the alpha band are significantly affected by corticocortical synaptic loss^16^,^17^. Secondly, cognitive decline in aMCI has been associated with anomalies in the power (or phase synchrony) of posterior alpha sources during both conditions of resting state^18^,^19^ and memory encoding^20^. And thirdly, there is evidence that abnormal alpha EEG oscillations are a functional reflection of cortical/subcortical atrophy across the disease^21^,^22^,^23^,^24^,^25^, and particularly of the hippocampal^26^ and amygdalo-hippocampal complex atrophy^27^, so prediction is expected to improve if atrophy of MTL structures is incorporated into the model.

## Materials and Methods

### Subjects

Sixty subjects were recruited from senior’s citizen associations, normal community health screening, and hospital outpatient services. All participants were right-handed. Twenty-six were cognitively normal controls and 34 met core clinical criteria for aMCI due to Alzheimer’s disease with an intermediate level of certainty^28^. Individuals with aMCI were divided into an “aMCI-converter” group (aMCI-c) that progressed to AD within a 2-year follow-up (N = 16), and an “aMCI-stable” group (aMCI-s) that remained stable during this time period (N = 18). Before participation, they gave informed consent to the experimental protocol approved by the Ethical Committee for Human Research at the Pablo de Olavide University according to the principles outlined in the Declaration of Helsinki. The methods were carried out in accordance with the approved guidelines. Inclusion and exclusion criteria for each one of these groups are described in Supplementary Material 1.

### Structural MRI acquisition and processing

Two 3D T1-weighted magnetization-prepared rapid gradient echo (MP-RAGE) images (repetition time = 8.5 ms, echo time = 4 ms, flip angle = 8°, matrix dimensions 256 × 192, 184 contiguous sagittal 1.2-mm-thick slices, and time per acquisition = 5.4 min) were acquired on a whole-body Philips Intera 1.5-T MRI scanner (Philips, The Netherlands), and were averaged after motion correction.

Cerebral MRI data were preprocessed using Freesurfer v5.3 (http://surfer.nmr.mgh.harvard.edu/). Removal of non-brain tissues was manually performed on a slice-by-slice basis in each participant to increase the accuracy of segmentation. Volumetric measures (mm^3^) were obtained for left and right sides of the hippocampus and amygdala, two MTL regions that have shown reduced volume in aMCI patients that progressed to AD at 1-year follow-up^29^.

### Experimental paradigm to evaluate associative memory

The experimental paradigm used to assess associative memory is described in detail elsewhere^30^. Briefly, during the encoding task, participants were instructed to indicate whether or not the face (famous or non-famous), presented in one of four possible locations, matched the biographical cue presented immediately before in the same location. The biographical cue could be semantically congruent or incongruent with the face. Next, subjects performed a conceptual priming task, where all faces were presented in the same spatial location as in the encoding task, but they were not preceded by any biographical cue. Participants were asked to identify as accurately and quickly as possible whether a face corresponded to a famous or non-famous person. Finally, faces were presented again without any preceding cue in the memory task, either at the same location as in previous phases or at any of the three remaining locations. For each face, participants were required to identify as quickly and accurately as possible whether or not they matched the previous location.

### Behavioral measures

Measures of recognition accuracy (*d’*) were computed not only for semantically congruent faces (SCF) and semantically incongruent faces (SIF) separately, but also for the combination of these two conditions to obtain a measure of global episodic memory for famous faces (*associative d’*). We also computed an index that quantified the congruency benefit as revealed by differences in *d’* between semantically congruent and incongruent faces (*semantic d’*). In all cases, *d’* resulted from subtracting the z-score for the false alarm rate from the z-score for the hit rate. We further evaluated reaction times (RT) for correct responses to intact and rearranged face-spatial location associations (hits and correct rejections, respectively).

### EEG recordings and signal preprocessing

EEG recordings were obtained from 59 scalp electrodes referenced to linked-mastoids, and positioned according to the extended International 10–20 system. Additional electrodes were placed for monitoring vertical-horizontal eye movements and the muscular tone. Skin-electrode impedances were maintained below 5 KΩ in EEG sensors. Ocular and muscle artifacts were partially removed from EEG recordings by applying Independent Component Analysis (ICA, Infomax algorithm) as implemented in the BrainVision Analyzer software v. 1.05 (Brain Products^®^ GmbH). The remaining noisy epochs were manually selected and excluded from further analyses. Artifact-free epochs were then transformed into the common average reference. As participants are expected to recognize a higher number of congruent than incongruent trials, differences in EEG oscillations could be partially due to differences in the signal-to-noise ratio. To counteract this effect, we included in the analysis an identical number of trials in each congruence condition for each subject, provided that they were correctly recognized in at least two trials during the memory task. The mean number of trials ± standard deviation was 55.7 ± 12.8 for HO, 46.1 ± 15.0 for aMCI-s, and 38.8 ± 12.5 for aMCI-c.

### Time-frequency representation of EEG data

The Fieldtrip toolbox (http://fieldtrip.fcdonders.nl/) was used to calculate baseline-corrected time-frequency representations (TFR) of spectral power across trials for each semantic condition and group. As growing evidence suggests that brain oscillations below 20 Hz are one of the core mechanisms of episodic memory^31^, we analyzed spectro-temporal information of frequencies ranging from 2 to 25 Hz by using a Morlet wavelet of 6 cycles. Next, we obtained task-related TFR of power decreases/increases relative to baseline defined from −2100 to −1600 ms and from −600 to −100 ms with respect to face onset during the encoding and memory task, respectively. When transformed to percentage, these normalized power values are equivalent to the event-related desynchronization/event-related synchronization (ERD/ERS) measure^32^.

### Statistical analysis

#### Behavior

Statistical analysis of behavioral data was conducted using SPSS software package (Version 22.0; SPSS inc., USA). Group differences in gender were assessed with the Chi-square test while the remaining variables were tested using one-way analyses of variance (ANOVAs) with group (HO, aMCI-s, and aMCI-c) as the between-subjects factor. Results derived from the memory task were evaluated with a mixed analysis of covariance (ANCOVA) including group as the between-subjects factor, semantic cue (SCF and SIF) as the within-subjects factor, and age as nuisance. Post-hoc comparisons were performed with the Bonferroni correction.

#### MRI volume of the hippocampus and amygdala

Mixed ANCOVAs with group as the between-subjects factor, hemisphere as the within-subjects factor, and age and total intracranial volume (ICV) as nuisance variables were performed to identify group differences in the volume of the hippocampus and amygdala.

#### EEG oscillations

We first computed the partial-least square (PLS) at the sensor level to identify the strongest statistical effects in frequency, time, and space domains simultaneously, by using the PLScmd Matlab toolbox (http://www.rotman-baycrest.on.ca/pls/). This multivariate statistical technique attempts to explain the covariance between two blocks of explanatory (i.e., contrasts or memory indices like *associative* and *semantic d’*) and dependent variables (i.e., normalized EEG activity) by a small number of uncorrelated variables known as latent vectors. This approach was employed to evaluate: i) differences in EEG oscillations with respect to the baseline period, ii) differences in EEG oscillations between HO and aMCI, ii) differences in EEG oscillations between aMCI-s and aMCI-c, and iv) the relationship between behavioral data in the memory task and EEG oscillations. The whole statistical procedure is explained in detail in Supplementary Material 2.

Cortical EEG sources were only estimated in those spectro-temporal windows showing significant group differences (HO vs. aMCI or aMCI-s vs. aMCI-c) and/or significant increases/decreases of normalized power with respect to the baseline period. To this aim, we applied a beamforming approach with multiple constraints adapted to each individual data (see Supplementary Material 3). Further details related to the statistical approach employed at the source level are described in Supplementary Material 4.

#### Regression analysis to predict conversion from aMCI to AD

Regression analysis included only MRI volumes and/or EEG measures, either showing significant differences between aMCI-s and aMCI-c, or significant correlations with performance that distinguished the aMCI groups.

For the EEG measures, we first created spheres with a radius of 3 voxels, the largest radius that allowed us to avoid inclusion of non-significant values in the sphere while maintaining the voxel showing the local maxima as center of mass after either comparing the two groups or after correlation with performance for alpha and beta power during both the encoding and memory task. Next, results of voxels comprising each sphere were averaged. And finally, we averaged results from different spheres belonging to the same functional area (e.g., left posterior cingulate cortex).

For each frequency band (i.e., alpha and beta) and task (i.e., encoding and memory), separate forward stepwise logistic regression analyses, based on the likelihood ratio, were first conducted for clustered regional EEG measures derived from different contrasts. The same analysis was applied for the total volume of the hippocampus and amygdala, including the ICV as nuisance variable.

All models were cross-validated with a “leave-10-out” approach, by using 90% of the sample as the training set, and the remaining 10% as the validation set. To reduce variability, multiple rounds (N = 34) of cross-validation were performed, and the validation results were averaged over the rounds. The main outcome measure was the averaged area under the curve (AUC) from the receiver-operating characteristic (ROC) curve with their corresponding confidence intervals. Additionally, we computed averaged overall accuracy, sensitivity, and specificity based on the cutoff value maximized with the Youden index (sensitivity + specificity − 1). The statistical significance of validation results was assessed with permutation testing (N = 10,000).

Every MRI volume that survived the previous analysis was included in an independent logistic regression analysis with each one of the EEG variables whose predictive value was above the 95^th^ percentile of the permutation distribution. In these particular cases, the ICV was also included as nuisance variable. All these models were also subjected to cross-validation and permutation testing. Finally, to determine which of these MRI, EEG, and MRI + EEG statistical models were better in predicting conversion to AD, we applied the Friedman test and the Wilcoxon signed-rank test with Bonferroni correction on the AUC and accuracy scores derived from the cross-validated models. For those models including more than one predictor variable, we determined the relative contribution of each predictor by applying the relative weight analysis, and calculated bootstrapped confidence intervals with the bias corrected accelerated method to assess whether the contribution of each predictor was significantly greater than zero.

## Results

### Demographic and cognitive profile

Table 1 shows demographic and cognitive data for each group at baseline. The three groups were statistically homogeneous in age, gender, and education years. As expected, a main effect of group was found for all cognitive measures including the MMSE (*F*_1,57_ = 4.4, *p* < 0.02), immediate (*F*_1,57_ = 21.7, *p* < 10^−7^), and delayed memory (*F*_1,57_ = 41.4, *p* < 10^−11^), the latter two scores derived from the Spanish version of the Logical Memory subtest extracted from the Wechsler Memory Scale-Third Edition^33^. These differences remained significant after adjusting by age and were mostly due to the better performance shown by HO, although aMCI-c also showed poorer delayed memory when compared with aMCI-s (*p* < 0.002).

### Behavioral results in the associative memory task

**Figure 1:**
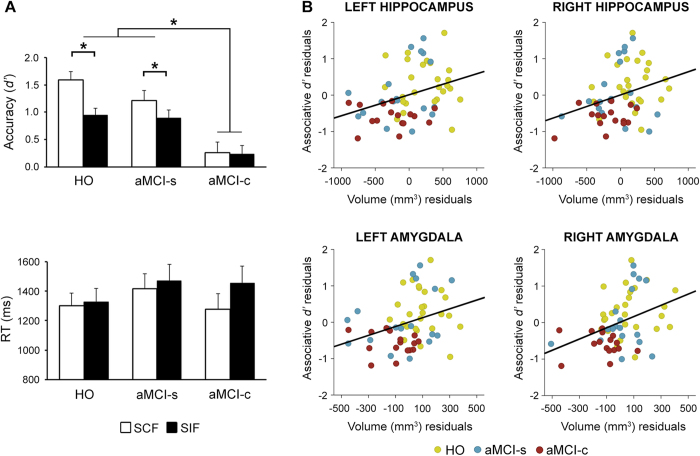
Memory performance in the recognition task and associations with hippocampal and amygdala volumes. (**A**) Mean accuracy and RT in the face-location memory task. Vertical lines on the bars refer to mean standard error. Asterisks indicate significant differences between the two conditions of congruency and between the aMCI-c group and the remaining two groups. (**B**) Scatter plots depicting correlation between residuals of *associative d’* and residuals of hippocampal or amygdala volume either on the left or right hemisphere after regressing these variables on age and total ICV. Each color represents one group. Given that regressions only reached significance when all participants were included in the analysis, only one fitted regression line was shown for each scatter plot.

The ANCOVA revealed no significant main effect of congruence for accuracy, but it showed a main effect of group (*F*_2,56 = _10.8; *p* < 0.0001) and a significant congruence × group interaction (*F*_2,56  _ = 7.1; *p* < 0.002). As expected, aMCI-c exhibited poorer memory recognition than aMCI-s (*p* < 0.001) and HO (*p* < 0.0001), apart from being the only group that did not benefit from semantic congruence (Fig. 1A, top panel). Regarding RT data, no main effect of congruence, group, or congruence × group interaction was observed (Fig. 1A, bottom panel).

The absence of congruency benefit shown by the aMCI-c group was unlikely due to a deficiency in automatically accessing semantic information, as revealed by results derived from the conceptual priming task. HO were more accurate than aMCI-c (*p* < 0.01) as suggested by the main effect of group (F_2,56_ = 5.4; *p* < 0.05). However, the main effect of congruence on both accuracy (F_1,57 _ = 127.3; *p* < 10^−15^) and RT (F_1,57 _ = 26.4; *p* < 10^−5^), and the lack of congruence × group interaction indicated that all three groups were more accurate and faster in response to SCF as compared with SIF.

The worst performance shown by the aMCI-c group during retrieval was also unlikely due to deficiencies in understanding task instructions. Two additional results support this idea. Firstly, no group differences were found regarding the percentage of omissions (times that people gave no response, HO = 0.1%, aMCI-s = 2.7%, aMCI-c = 1%). And secondly, although the aMCI-c group showed the lowest percentage of successful performance (proportion of hits plus correct rejections) during retrieval (F_2,56_ = 11.6; *p* < 10^−4^), we found no significant group differences in the percentage of correct responses during the encoding (F_2,56_ = 2.4; *p* = 0.1) and conceptual priming task (F_2,56_ = 2.9; *p* = 0.06).

### Volume of hippocampus and amygdala: group differences and relationship with memory

The ANCOVAs yielded significant group differences for the volume of the hippocampus (*F*_2,54_ = 11.4; *p* < 0.0001) and amygdala (*F*_2,54_ = 9.6; *p* < 0.0003). For both MTL structures, HO showed higher volume than any of the aMCI groups (0.0001 < *p* < 0.05). However, only for the amygdala, aMCI-c showed greater atrophy than aMCI-s (*p* < 0.03). The mean and standard deviation values of these two MTL regions in the left and right hemisphere for each group are shown in Table 1.

Regression analyses across all participants revealed a positive correlation between *associative d’* and the volume of hippocampus and amygdala in the left (0.44 < *β* < 0.51) and right hemisphere (0.0002 < *p* < 0.00007). These relationships did not achieve statistical significance when regression was performed within each group separately. Interestingly, significance was only lost when the aMCI-c group was removed from analyses but not when one of the two remaining groups was removed. This finding highlights the remarkable contribution of the aMCI-c group to this relationship, and indicates that the associative deficit becomes evident when the MTL reaches a certain level of atrophy. The regression analysis with *semantic d’* only survived Bonferroni correction for the left hippocampus (*β* = 0.36; *p* = 0.006). The scatter plots of the relationship between the residuals of associative memory and volume of each MTL region after regressing these variables on age and total ICV are depicted in Fig. 1B.

### EEG oscillations

Results at the sensor level are described in detail in Supplementary Material 5. Briefly, HO showed a significant decrease of beta power (i.e., higher beta ERD) compared with aMCI during both the encoding (14–25 Hz; 0–1000 ms; *p* < 0.04) and retrieval task (13.5–18 Hz; 700–1000 ms; *p* < 0.05). During memory recognition, aMCI-s further showed higher alpha ERD than aMCI-c (see Figs S1 and S2, respectively, in Supplementary Material 5).

The EEG sources responsible for these differences were estimated for 8.5 Hz within 500 and 1000 ms during both the encoding and retrieval phases, for 15.75 Hz between 400 and 900 ms during encoding, and for 16.25 Hz between 500 and 1000 ms during retrieval.

### Changes in alpha/beta ERD associated with AD progression

Although changes in alpha/beta oscillations distinguished aMCI-c from the remaining groups, and the aMCI-s group could further be distinguished from HO on the basis of the relationship between these oscillations and different aspects of memory (see Figs S2, S3 and Tables S1–S4 in Supplementary Material 6), here we will specifically focus on those aspects that allowed us to discriminate aMCI-s from aMCI-c, since they are the only ones that will be used in prediction analyses.

**Figure 2:**
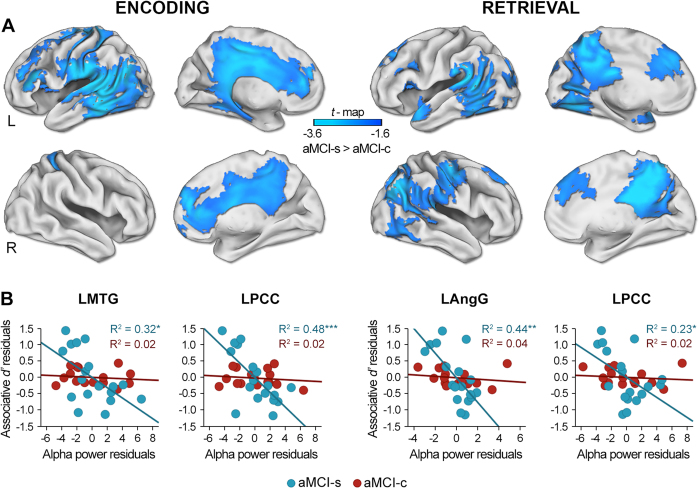
Topographic distribution of group differences in alpha ERD and correlations with memory performance. (**A**) Statistical nonparametric maps showing higher alpha ERD in aMCI-s than in aMCI-c during encoding and retrieval. (**B**) Scatter plots illustrating the relationship between residuals of alpha ERD in a voxel showing local maxima in the group contrast and residuals of memory performance in the recognition task (*associative d’*), for both aMCI-s and aMCI-c after regressing these variables on age. L = left; R = right; LMTG = left middle temporal gyrus; LPCC = left posterior cingulate; LAngG = left angular gyrus; *p < 0.05; **p < 0.005; ***p < 0.001.

During encoding, aMCI-c showed decreased alpha ERD with respect to aMCI-s (*p*_*cluster*_ < 0.02) on a widespread cortical network comprising medial aspects of bilateral frontal and parietal lobes as well as regions in the left lateral and medial regions of the temporal lobe (Fig. 2A and Table 2). The aMCI-c group also showed lower alpha ERD (*p*_*cluster*_ < 0.03) as compared with the aMCI-s group in most of the regions mentioned above during the memory task. These differences further extended to posterior lateral regions of the right hemisphere. Posterior cortical regions of the alpha network that distinguished aMCI-s from aMCI-c during both encoding and retrieval were correlated with *associative d’* only in the aMCI-s group (see Table S5 in Supplementary material 7). This finding is illustrated in the scatter plots depicted in Fig. 2B for encoding and retrieval, for both aMCI-s and aMCI-c individuals. Note that alpha power in the left posterior cingulate cortex during encoding showed the strongest contribution to regression in the aMCI-s group (R^2^ = 0.48; *p* = 0.001).

While encoding associative memories, the aMCI-c group further showed decreased beta ERD (*p*_*cluster*_ < 0.006) with respect to the aMCI-s group over the anterior cingulate bilaterally, left inferior parietal lobe, and some lateral regions of the right frontal lobe. However, these differences were restricted to the right parahippocampal gyrus during retrieval (Table 3). Unlike alpha oscillations across cortical networks, beta activity in these brain regions was unrelated to memory performance.

### Group differences in the relationship between beta ERD and memory

**Figure 3:**
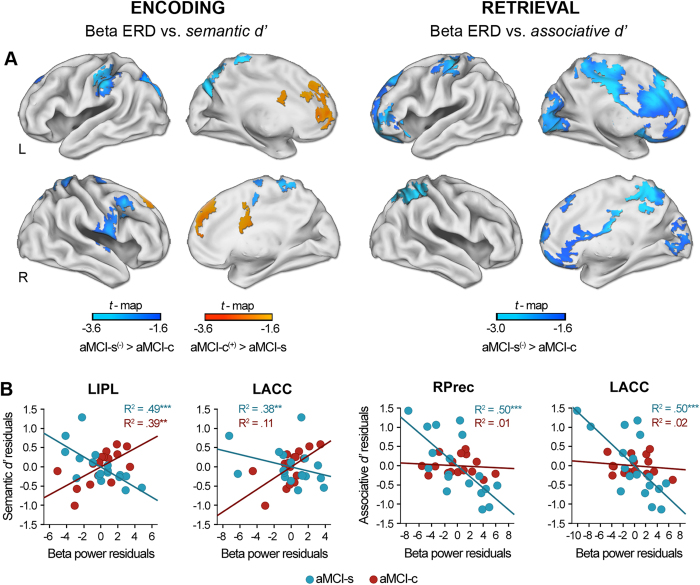
Group differences in correlations between memory and beta ERD. (**A**) Statistical nonparametric maps showing significant differences between aMCI-s and aMCI-c in correlations between beta ERD and memory performance (*semantic d’* and *associative d’)*. Blue areas indicate that the higher the beta ERD over these regions the better the memory performance in aMCI-s with respect to aMCI-c. Orange areas indicate that the higher the beta ERD over these regions the lower the benefit from semantic congruence in aMCI-c with respect to aMCI-s. (**B**) Scatter plots illustrating the relationship between residuals of beta ERD and residuals of memory performance in the recognition task in a voxel showing local maxima in the correlation, for both aMCI-s and aMCI-c after regressing these variables on age. L = left; R = right; LIPL = left inferior parietal lobe; LACC = left anterior cingulate; RPrec = right precuneus; *p < 0.05; **p < 0.005; ***p < 0.001.

Differences in beta ERD between SCF and SIF during encoding were more strongly correlated with the benefit of semantic congruence in aMCI-s than in aMCI-c over the left inferior parietal lobe (*p*_*cluster*_ < 0.02), right superior parietal lobe (*p*_*cluster*_ < 0.003), and right middle frontal, precentral, and postcentral gyrus (*p*_*cluster*_ < 0.02) (Fig. 3A and Table 4). On the contrary, the aMCI-c group showed a stronger correlation compared to aMCI-s over areas of the medial prefrontal cortex. In this particular case, the higher the activation in these regions in response to SCF with respect to SIF, the lower the benefit of congruence shown by the aMCI-c group in the memory task.

During retrieval, aMCI-s showed a significantly stronger correlation between beta ERD and associative memory in a widespread cortical network when compared with aMCI-c (*p*_*cluster*_ < 0.04) (Fig. 3A, and Table 4). These differences were firstly evident in the posterior cingulate gyrus bilaterally and in the dorsal motor regions of the left hemisphere (from 252 ms until the end of the analysis interval), and further extended to the right superior parietal lobe at about 400 ms and to the inferior parietal and frontal lobe from 500–600 ms onward. Figure 3B displays the scatter plots of group differences in the regression slopes between aMCI-s and aMCI-c for a representative voxel in the left anterior cingulate and right precuneus.

### Predicting conversion from aMCI to AD

The forward stepwise logistic regression analysis derived from MRI (i.e., volume of hippocampus and amygdala) and based on the likelihood ratio test reached statistical significance only when the right amygdala was introduced into the model (χ^2^ = 9.2, *p* < 0.01). After conducting similar analyses for each frequency band (i.e., alpha and beta), only two models integrated by one alpha-generating source each were significant: one during encoding and the other during retrieval. Both alpha sources not only distinguished aMCI-s from aMCI-c, but they were also negatively correlated with the *associative d’* index in aMCI-s (Fig. 2B). In particular, the model derived from the encoding task included the alpha ERD over the left posterior cingulate (PCC, BA23 and BA31) (χ^2^ = 14.2, *p* < 0.0002), whereas the one resulting from the memory task included the alpha ERD over the left cuneus/precuneus (BA17 and BA7) (χ^2^ = 11.4, *p* < 0.001). The logistic regression analysis also revealed significant models when the volume of the right amygdala was combined with the alpha ERD during encoding (χ^2^ = 18.9, *p* < 0.0003) and retrieval (χ^2^ = 15.9, *p* < 0.001). Table 5 contains results associated with each one of these single models as well as with the composite models including MRI and EEG measures after applying cross-validation and permutation testing.

The Friedman test was significant for overall accuracy (χ^2^ = 100.8, *p* < 10^−20^), AUC (χ^2^ = 123.4, *p* < 10^−25^), sensitivity (χ^2^ = 75.3, *p* < 10^−14^), and specificity (χ^2^ = 76.5, *p* < 10^−15^), suggesting that some models were better predictors of AD progression than others. The post hoc Wilcoxon signed-rank tests yielded significant differences between almost all of the models after Bonferroni correction (*p* < 0.002). Among the exceptions, the single model with the amygdala was found to improve neither the overall accuracy nor the specificity of the single model including the left PCC alpha ERD. Even so, the combination of right amygdala volume and alpha power over the left PCC showed the highest prediction capability. The relative weight was 67.7% for the left PCC and 24% for the amygdala, but only contribution of the former achieved statistical significance (CI = [0.04 1690.80]). In the model including the left cuneus/precuneus the relative weight analysis yielded similar results. The amygdala explained 26.7% of the variance and the left cuneus/precuneus the 61.1%, only the latter being significantly different from zero (CI = [0.02 114.47]).

## Discussion

The present study revealed that aMCI-c at baseline, besides showing the lowest scores in associative memory were unable to benefit from semantic congruency. These memory deficits were associated with volume loss of hippocampus and amygdala and with a reduced capacity to engage control processes and to reactivate context-specific sensory information through desynchronizing mechanisms in the alpha/beta band during both encoding and memory retrieval. In consonance with these findings, the combination of MRI data with abnormal memory-related EEG oscillations provided the highest accuracy in predicting overt dementia. Particularly, atrophy of the right amygdala together with encoding-induced anomalies in alpha oscillations over the left PCC correctly classified aMCI-s and aMCI-c with a sensitivity of 78% and specificity of 82%, and with an overall accuracy of 81%.

Although the hippocampus has traditionally captured more attention than the amygdala, both structures are critically involved in memory processes and present a similar degree of volume loss in AD^34^,^35^. Nevertheless, it remains to be determined whether amygdala atrophy, like hippocampal atrophy^36^, accounts for the emotional changes occurring with disease progression. We found a higher degree of amygdala atrophy in aMCI-c than in aMCI-s, suggesting that the amygdala could be considered as a marker of dementia severity. Accordingly, reduction of the amygdala volume has been previously reported to predict the risk of progression to AD with a sensitivity of 76% and specificity of 68%^37^. The present study suggests that the prediction accuracy of amygdala atrophy can be significantly improved by including in the model alterations in task-related EEG oscillations associated with memory deficits.

The relationship between alpha desynchronization and successful encoding/retrieval of episodic memories is well documented^38^. We believe that anomalies in the amplitude of alpha oscillations over PCC in parallel with memory deficits in aMCI-c may be a reflection of the early synaptic alterations in this particularly vulnerable region. In line with this idea, a postmortem tissue study found synaptic loss in the PCC of MCI accompanied by a decline of different synaptic markers, all of which were significantly associated with lower MMSE scores and with increased uptake of the amyloid PET tracer ^11^C Pittsburgh compound B^39^. These synaptic alterations in the PCC might also account for the association between early Aβ deposition and aberrant fMRI activity^40^ as well as for abnormal local and long-range synchronization of EEG/MEG patterns during the resting state in MCI patients^19^,^41^,^42^,^43^,^44^. For instance, in one of these studies, MCI patients with reduced CSF Aβ42 levels showed decreased functional connectivity in the alpha band between the right PCC and lateral regions of the temporal cortex^19^.

Likely, the neurotoxic effects of Aβ are not solely responsible for abnormal EEG oscillations in aMCI. In fact, reduction of microtubules through tau hyperphosphorylation and tau aggregation might also lead to disruption in the transport of molecules and organelles to and from the synapse, thereby resulting in synaptic dysfunction and axonal degeneration^45^. Thus, the above-mentioned MEG study^19^ further reported decreased alpha synchronization in the right PCC associated with increased levels of CSF phosphorylated tau.

Finally, anomalies in memory-related EEG oscillations could also be partially due to disruption of fast synaptic inhibition mediated by γ-aminobutyric acid (GABA) neurons. Extensive research suggests that GABAergic neurons play a critical role in generating network synchrony in general^46^ and in the alpha band in particular^47^. In agreement with this hypothesis, MCI showed lower GABA levels in the PCC than HO in parallel with memory deficits^48^.

The amygdala and PCC are anatomically^49^,^50^ and functionally^51^ connected via the anterior cingulate/medial prefrontal cortex, and via the retrosplenial cortex and parahippocampal gyrus. Interestingly, all these cortical regions not only were associated with successful memory retrieval in aMCI-s, but they also showed abnormal alpha oscillatory activity in aMCI-c. Evidence further suggests that the amygdala and PCC are anatomically^52^ and functionally^53^ connected to the hippocampus, one of the first regions affected in AD. In fact, we have demonstrated here and in previous studies^54^ that hippocampal volume loss is associated with impaired memory performance in the face-location memory task. Finally, it is well known that both the amygdala and PCC receive cholinergic projections from the basal forebrain complex, mainly from the nucleus basalis of Meynert^55^. This nucleus is severely affected by neurofibrillary degeneration and cell loss in AD^56^, supporting recent evidence showing a selective relationship between reduction of this cholinergic nucleus and amygdala atrophy in aMCI^57^. Collectively, evidence reviewed here suggests that a dysfunction and loss of basal forebrain cholinergic neurons together with synaptic and axonal alterations affecting amygdala, PCC, and PCC fiber tracts, likely account for the fact that amygdala atrophy and abnormal alpha oscillatory activity over PCC provided the most accurate prediction of aMCI to AD conversion.

Unlike MRI, cognitive and EEG measures revealed no differences between HO and aMCI-s individuals. However, the relationship between memory and neural oscillatory activity was stronger in aMCI-s than in HO, which points to the need for additional neural and cognitive resources in this population. In particular, associative memory in the aMCI-s group depended to a great extent on the frontoparietal network, while the benefit of congruency was associated with higher activation of the precuneus. Both the frontoparietal network^58^ and the precuneus^59^ exhibit connectivity with a variety of neural networks depending on current task demands. If oscillatory EEG changes observed in these brain systems serve a compensatory function, longer longitudinal studies will have to determine whether overuse of these regions make them more vulnerable to AD lesions, and consequently to dementia.

## Conclusions

The PCC is considered a major cortical hub in the human brain because it maintains dense structural connectivity to widespread regions involved in multiple memory functions and control processes^60^. Together with limbic structures like amygdala and hippocampus, PCC is one of the cortical regions most vulnerable to AD. The present study reinforces this idea. Indeed, we showed that the higher the EEG alpha desynchronization over PCC, the better the memory in aMCI-s, and that failures in this desynchronizing mechanism predicted AD conversion from aMCI better than amygdala atrophy. Nevertheless, the best predictive model was obtained when alpha oscillations over PCC were combined with volume loss of the amygdala, which highlights the importance of combining electrophysiological correlates of synaptic activity with MRI biomarkers of AD to anticipate dementia.

Although these results are promising, they must be considered as preliminary. Firstly, because the sample was not very large and over-fitting may still be present in spite of cross-validation. Secondly, because logistic regression was applied to particular features that had been previously selected by analyzing the statistical characteristics of that particular sample and this can limit generalization capability. And thirdly, because two years is a relatively short follow-up period, and new conversions are expected to occur at a similar rate in the following years. Consequently, future research is needed in order to validate the present model in an independent, larger sample including a longer follow-up period.

## Additional Information

**How to cite this article**: Prieto del Val, L. *et al.* Atrophy of amygdala and abnormal memory-related alpha oscillations over posterior cingulate predict conversion to Alzheimer’s disease. *Sci. Rep.* **6**, 31859; doi: 10.1038/srep31859 (2016).

## Supplementary Material

Supplementary Information

## Figures and Tables

**Table 1:** Demographic data and cognitive profile.

	HO (n = 26)	aMCI-s (n = 18)	aMCI-c (n = 16)	*p<*
Age, yr	66.7 ± 4.9	68.4 ± 7.1	69.7 ± 6.5	0.3
Gender (F/M)	13/13	13/5	9/7	0.4
Education, yr	7.3 ± 4.4	7.3 ± 5.1	7.7 ± 6.3	0.9
CDR	0	0.5	0.5	N/A
MMSE	28.4 ± 1.3	26.8 ± 2.1	26.4 ± 2.7	0.02
Immediate recall	14.3 ± 3.1	10.5 ± 2.3	8.9 ± 2.7	10^−7^
Delayed recall	13.2 ± 2.9	8.3 ± 3.4	4.5 ± 2.9	10^−11^
L Hippocampal volume	3220.8 ± 303.4	2837.0 ± 563.5	2556.6 ± 483.7	10^−4^
R Hippocampal volume	3231.9 ± 355.5	2981.4 ± 481.0	2701.2 ± 333.5	0.001
L Amygdala volume	1437.9 ± 97.4	1280.3 ± 281.6	1169.9 ± 178.9	0.001
R Amygdala volume	1459.2 ± 164.6	1395.8 ± 229.6	1196.4 ± 155.7	0.001

Results are expressed as mean ± SD (standard deviation). F/M = female/male. CDR = Clinical Dementia Rating (0 no dementia, 0.5 questionable or very mild dementia). MMSE = Mini-Mental State Examination. N/A = no applicable; *p* = *p*-value indicating a main effect of group (for hippocampal and amygdala volume, age and intracranial volume were introduced as nuisance).

**Table 2:** Cortical regions showing group differences in alpha ERD during the encoding and retrieval task.

Cortical region	BA	*x*	*y*	*z*	*t*	Time (ms)	*T*	*p*
**ENCODING TASK**								
**aMCI-s > aMCI-c** *p*_cluster_ < 0.02								
L Superior temporal gyrus	22	−55	−43	13	−3.62	208–980	−4.24/−2.31	0.0002–0.03
L Middle temporal gyrus	20	−65	−48	−17	−3.62	0–980	−4.36/−2.26	0.001–0.02
L Inferior parietal lobe	40	−65	−28	28	−3.53	208–980	−4.70/−2.44	0–0.02
L Postcentral gyrus	5	−30	−33	73	−3.40	0–980	−4.27/−2.29	0.0002–0.03
R Postcentral gyrus	7	15	−53	78	−2.25	624–884	−2.39/−2.31	0.02
L Anterior cingulate gyrus	32	0	42	23	−3.34	468–980	−4.93/−2.47	0–0.02
L Anterior cingulate gyrus	24	−10	−8	38	−3.26	0–980	−3.95/−2.29	0.0007–0.03
L Middle frontal gyrus	46	−50	32	18	−3.30	0–980	−4.51/−2.09	0.0001–0.05
L Posterior cingulate gyrus	31	−10	−28	48	−3.20	0–980	−3.62/−2.72	0.001–0.02
R Posterior cingulate gyrus	23	5	−13	28	−2.73	0–980	−3.59/−2.90	0.001–0.006
L Precentral gyrus	4	−25	−23	58	−2.98	312–980	−3.51/−2.19	0.002–0.04
R Medial frontal gyrus	9	10	47	28	−2.94	572–980	−4.91/−2.61	0–0.02
L Medial frontal gyrus	10	0	72	−2	−2.70	572–980	−3.28/−2.42	0.002–0.03
L Transverse temporal gyrus	42	−65	−13	8	−2.80	416–980	−3.21/−2.32	0.003–0.03
L Superior parietal lobe	7	−20	−58	73	−2.22	520–980	−2.64/−2.41	0.01–0.02
**RETRIEVAL TASK**								
**aMCI-s > aMCI-c** *p*_cluster_ < 0.03								
L Superior temporal gyrus	22	−65	−48	23	−3.65	252–980	−4.88/−2.33	0–0.03
L Temporal pole	38	−55	7	−12	−3.01	356–980	−2.69/−2.21	0.01–0.03
R Precuneus	7/19	5	−48	38	−3.59	252–980	−3.88/−2.53	0.0005–0.02
R Postcentral gyrus	3	50	−18	38	−3.11	252–876	−4.43/−2.37	0.0001–0.03
R Middle temporal gyrus	39	35	−58	28	−3.00	408–980	−3.26/−2.57	0.002–0.02
L Middle temporal gyrus	21	−70	−48	−12	−2.45	460–980	−2.81/−2.38	0.008–0.02
R Superior parietal lobule	7	15	−68	68	−2.99	356–980	−3.48/−2.34	0.001–0.03
L Anterior cingulate gyrus	32	−10	32	43	−2.92	460–980	−3.19/−2.57	0.003–0.02
L Cuneus	18/19	−15	−93	33	−2.45	460–928	−2.54/−2.39	0.01–0.02
L Insula	13	−45	17	13	−2.21	512–772	−2.45/−2.39	0.01–0.02
L Superior frontal gyrus	8	−15	47	48	−2.75	564–980	−2.69/−2.65	0.01
R Superior frontal gyrus	6	25	42	53	−2.48	512–928	−2.56/−2.37	0.01–0.02
L Medial frontal gyrus	9	0	42	28	−2.51	668–876	−2.61/−2.58	0.01
R Inferior parietal lobe	40	65	−23	38	−2.72	356–980	−3.41/−2.23	0.001–0.04
R Middle occipital gyrus	19	55	−73	−7	−2.65	512–928	−3.39/−2.14	0.02–0.04
L Angular gyrus	39	−55	−63	38	−2.63	356–876	−3.88/−2.05	0.0005–0.02
R Supramarginal gyrus	40	65	−53	28	−2.31	408–980	−2.80/−2.30	0.009–0.02
R Medial frontal gyrus	9	15	17	48	−2.27	668–772	−2.52/−2.61	0.01–0.02

The direction of the contrast indicates that the first group showed higher low-alpha ERD (i.e., higher decrease of alpha power) than the second group. BA = Brodmann area; L = left; R = right; *t* = *t*-statistic at the source level; *T* = range of *t*-statistics in the time domain; *p* = range of *p* values in the time domain.

**Table 3:** Cortical regions showing group differences in beta ERD during the encoding and retrieval task.

Contrast Cortical region	BA	*x*	*y*	*z*	*t*	Time (ms)	*T*	*p*
**ENCODING TASK**								
**aMCI-s > aMCI-c**								
*p*_cluster_ < 10^−5^								
R Superior frontal gyrus	6/8	35	2	68	−3.11	156–936	−2.41/−2.36	0.004–0.03
R Postcentral gyrus	2	40	−23	33	−2.87	260–676	−2.37/−2.32	0.02–0.04
L Anterior cingulate gyrus	24	0	2	28	−2.72	156–884	−2.39/−2.33	0.007–0.03
R Inferior parietal lobe	40	45	−28	33	−2.56	0–624	−2.28/−2.22	0.02–0.04
R Middle frontal gyrus	8	30	27	43	−2.54	624–780	−2.40/−2.28	0.02–0.03
R Precentral gyrus	6	35	2	28	−2.39	572–780	−2.42/−2.24	0.02–0.03
L Medial frontal gyrus	6	−5	12	53	−2.28	208–624	−3.19/−2.39	0.003–0.03
*p*_cluster_ < 0.003								
L Postcentral gyrus	2	−40	−18	33	−2.81	208–1000	−3.26/−2.28	0.002–0.05
L Superior temporal gyrus	41	−40	−28	13	−2.57	520–767	−2.50/−2.36	0.02–0.03
L Precentral gyrus	4	−35	−13	43	−2.57	416–728	−2.51/−2.36	0.02–0.03
L Middle frontal gyrus	6	−30	−8	43	−2.37	416–468	−2.30/−2.23	0.03–0.04
**RETRIEVL TASK**								
**aMCI-s > aMCI-c** *p*_cluster_ < 0.006								
R Parahippocampal gyrus	30	35	−48	8	−2.56	408–720	−2.58/−2.14	0.02–0.04

The direction of the contrast indicates that the first group showed higher low-beta ERD (i.e., higher decrease of beta power) than the second group. BA = Brodmann area; L = left; R = right; *t* = *t*-statistic at the source level; *T* = range of *t*-statistics in the time domain; *p* = range of *p* values in the time domain.

**Table 4:** Cortical regions showing group differences in correlations between *semantic d’* and beta ERD during the encoding task and between *associative d*’ and beta ERD during the retrieval task.

Contrast Cortical region	BA	*x*	*y*	*z*	*t*	Time (ms)	*T*	*p*
**ENCODING TASK** (***semantic d’ vs**. **beta ERD***)								
**aMCI-s**^(**−**)^ > **aMCI-c**								
*p*_cluster_ < 0.003								
L Postcentral gyrus	5	−10	−38	78	−3.74	624–884	−3.60/−3.11	0.005–0.04
R Postcentral gyrus	7	10	−48	78	−3.30	0–936	−3.38/−2.95	0.004–0.04
R Precuneus	7	30	−68	63	−2.81	572–1000	−3.46/−2.30	0.001–0.02
R Superior parietal lobule	7	25	−63	68	−2.76	572–1000	−2.83/−2.22	0.004–0.02
*p*_cluster_ < 0.005								
L Cuneus	7	−20	−78	38	−4.13	520–832	−4.37/−3.35	0.001–0.04
L Precuneus	19/7	−15	−78	48	−4.05	572–780	−4.16/−3.56	0.001–0.03
*p*_cluster_ < 0.002								
L Inferior parietal lobule	40	−55	−28	48	−3.85	520–1000	−4.34/−3.23	0.002–0.05
L Inferior parietal lobule	40	−35	−33	43	−2.93	0–624	−3.14/−2.13	0.03–0.05
R Middle frontal gyrus	6	50	7	43	−3.42	624–728	−3.30/−2.93	0.02–0.04
R Precentral gyrus	6	55	7	48	−3.30	572–728	−3.09/−2.78	0.03–0.05
R Postcentral gyrus	43	70	−3	13	−3.10	208–676	−3.14/−2.04	0.003–0.05
**aMCI-c**^(**+**)^ > **aMCI-s** *p*_cluster_ < 0.003								
L Cingulate gyrus	32	0	47	8	−2.71	624–676	−2.68/−2.63	0.02–0.03
R Superior frontal gyrus	6	15	42	48	−2.66	416–676	−3.07/−2.64	0.004–0.03
L Medial frontal gyrus	9	−10	37	28	−2.34	676–832	−2.65/−2.55	0.02–0.04
R Posterior cingulate gyrus	23	5	−8	33	−2.30	832–1000	−2.50/−2.36	0.03–0.05
L Anterior cingulate gyrus	24	−10	2	33	−2.22	624–832	−2.37/−2.14	0.03–0.05
**RETRIEVAL TASK** (***associative d’ vs**. **beta ERD***)								
**aMCI-s**^(**−**)^ > **aMCI-c** *p*_cluster_ < 0.04								
R Posterior cingulate gyrus	31	25	−43	43	−4.12	252–1000	−4.15/−2.92	0.002–0.04
L Superior frontal gyrus	6/8	−5	32	58	−3.32	512–1000	−3.79/−2.87	0.001–0.03
R Precuneus	7	10	−58	68	−3.03	356–720	−3.31/−2.69	0.008–0.05
R Superior parietal lobe	7	15	−63	68	−3.00	408–928	−3.01/−2.22	0.01–0.04
L Superior frontal gyrus	10/11	−25	52	−17	−2.95	616–1000	−3.77/−2.80	0.001–0.02
L Posterior cingulate gyrus	31	−5	−28	38	−2.92	252–1000	−3.98/−2.80	0.01–0.04
L Anterior cingulate gyrus	32	−10	27	43	−2.89	564–1000	−3.62/−2.78	0.001–0.02
L Paracentral lobule	6	0	−23	58	−2.78	252–928	−3.53/−2.60	0.008–0.04
L Inferior parietal lobule	40	−35	−33	63	−2.75	668–876	−2.94/−2.78	0–0.04
L Inferior frontal gyrus	47	−30	37	−7	−2.67	616–1000	−2.92/−2.45	0.02–0.05

The sign (−) indicates the presence of a significant negative correlation in that particular group, meaning that the lower the beta power, the higher the benefit of semantic congruence in that group. The sign (+) indicates just the opposite. The direction of the contrast indicates that the first group showed a stronger correlation than the second group. BA = Brodmann area; L = left; R = right; *t* = *t*-statistic at the source level; *T* = range of *t*-statistics in the time domain; *p* = range of *p* values in the time domain.

**Table 5:** Predictive values of each classifier for AD progression.

Classifier	AUC (CI)	ACC%	SE%	SP%
R amygdala	0.75 (0.52–0.89)^*^	77.5^*^	74.4	79.2
αERD_E_ L PCC	0.80 (0.60–0.92)^*^	81.3^*^	58.3	92.6^*^
αERD_R_ L cuneus	0.77 (0.57–0.90)^*^	79.6^*^	75.4	71.7
**R amygdala** + α**ERD**_**E**_ **L PCC**	**0**.**82** (**0**.**63–0**.**93**)^*^	**80**.9^*^	**77**.7^*^	8**2**.2^*^
R amygdala + αERD_R_ L cuneus	0.78 (0.56–0.90)^*^	80.0^*^	63.6	6.1^*^

AUC = area under the curve; CI = confidence interval; ACC = accuracy; SE = sensitivity; SP = specificity; αERD_E_ = alpha event-related desynchronization during encoding; αERD_R_ = alpha event-related desynchronization during retrieval; PCC = posterior cingulate cortex; L = left; R = right; ^*^significant after permutation testing (*p* < 0.05). Bold type indicates the best fit model.
