# Supplementary material for: Atrophy of amygdala and abnormal memory-related alpha oscillations over posterior cingulate predict conversion to Alzheimer’s disease
Source: Sci Rep. 2016 Aug 22;6:31859. doi: 10.1038/srep31859 (PMC4992828; doi:10.1038/srep31859)
Supplement: Supplementary Information [file srep31859-s1.pdf]

Atrophy of amygdala and abnormal memory-related alpha oscillations over posterior cingulate predict conversion to Alzheimer's disease

Laura Prieto del Val, Jose L. Cantero, Mercedes Atienza*

SUPPLEMENTARY MATERIAL

1. Inclusion/exclusion criteria to participate in the study

Inclusion criteria for HO were: (i) aged 50-80 years old; (ii) scores between 27 and 30 in the global cognitive status assessed using the Spanish version of the Mini Mental State Examination (MMSE) (Lobo et al., 1979); (iii) absence of cognitive impairment confirmed by neuropsychological testing; (iv) global score of 0 (no dementia) in the clinical dementia rating (CDR) (Hughes et al., 1982); (v) normal independence function, judged both clinically and by means of an interview for deterioration in daily living activities validated in the Spanish population (Böhm et al., 1998); and (vi) no presence of neurological or psychiatric illness. Depression was excluded in all three groups with the shorter version of the Geriatric Depression Scale (Yesavage et al., 1983).

Inclusion criteria for individuals with aMCI were: (i) aged 50-80 years old; (ii) MMSE scores between 24 and 30; (ii) subjective memory complaints corroborated by the informant; (iii) objective memory loss confirmed by the Spanish version of the Logical Memory subtest extracted from the Wechsler Memory Scale-Third Edition (Wechsler, 2004) (scorings 1.5 standard deviations below the age-appropriate mean); (iv) absence of impairment in cognitive areas other than memory as revealed by neuropsychological testing; (v) CDR global score of 0.5 (questionable dementia); (vi) normal independence function; (vii) no criteria for dementia; and (viii) no presence of neurological or psychiatric illness.

MRI was examined in all participants by a neurologist to rule out lesions such as territorial cerebral infarction, brain tumor, hippocampal sclerosis, and/or vascular malformations. Those participants with periventricular and/or deep white matter lesions,

as revealed by scores ≥ 2 on the Fazekas ischemic scale (Fazekas et al., 1987), were excluded from the study. Individuals with a history of stroke and/or significant cerebrovascular conditions, clinically significant sensory impairment, past or current alcohol abuse, or those consuming medication known to affect memory, were not allowed to participate. None of the participants were taking cholinesterase inhibitors, and/or psychiatric medication at the time of recruiting or during the study. The absence of secondary causes of cognitive deficits was assessed by laboratory tests including complete blood count, blood chemistry, vitamin B12/folate, and thyroid function.

The diagnosis of AD in the aMCI-c group was based on criteria established by the National Institute of Neurological and Communicative Disorders and Stroke and the Alzheimer's Disease and Related Disorders Association (NINCDS-ADRDA) and by the Diagnostic and Statistical Manual of Mental Disorders IV (DSM IV). These subjects should further present MMSE scores ranging from 12 to 28 and a CDR global score of 1 (mild dementia). Subjects were excluded from the analysis if any psychiatric or neurological illness other than AD was present, and if subjects presented with a systemic illness or signs of organ failure.

2. Partial Least Square (PLS) analysis at the sensor level

We applied permutation tests to determine whether the effect of each latent variable reached statistical significance. For instance, to evaluate group differences in EEG oscillations (HO vs. aMCI; aMCI-s vs. aMCI-c), subjects were randomly reassigned 10,000 times without replacement to different experimental conditions (HO or aMCI; aMCI-s vs. aMCI-c), whereas for comparisons with the baseline period we permutated 10,000 times the pre- and post-stimulus period of interest within each subject. For testing correlations, the behavioral indices (i.e., *associative d'* and *semantic d'*) were permutated across subjects. Finally, we computed 2000 bootstrap estimations of the standard errors of the weights of the original variables to determine the significance of nonzero weights for the variables of the corresponding latent vector.

Within each time-frequency window where the PLS yielded significant results, we performed *t*-tests for related samples in the case of comparisons with baseline, *t*-tests for independent samples in the case of group differences, and Spearman correlations to evaluate the relationship between performance and the EEG power averaged over

significant sensors. Unlike Pearson correlations, Spearman correlations are quite robust to outliers and do not require that the joining distribution be multivariate normal. To correct the family-wise error (FWE) rate for all time point comparisons, the maximum-statistic approach was employed (Maris, 2004). For every time sample after the onset of the face-location association, we computed the corresponding statistic. This was repeated for each data randomization (N=1000), selecting the maximum (or minimum) statistic across all samples. The 95th quartile of the distribution derived from the selected maximum (or minimum) statistic was used as a critical threshold to retain or reject the null hypothesis of either no differences between experimental conditions or no correlation between normalized power and behavior.

3. Source modeling of EEG oscillations with beamforming

We first obtained from single trials at the sensor level the constant field distribution of non-phase locked oscillatory activity for the frequency bin of interest (Guderian and Düzel, 2005). To this aim, we calculated phase differences between sensor pairs at each time point of each single trial for the frequency bin showing the maximum statistic after averaging those sensors where the PLS yielded significant differences. This procedure calculates, for each subject, the phase lag index of all sensors with respect to the sensor with the maximum power. Next, we applied the inverse wavelet transformation to bring oscillatory amplitude information back to the time domain. Consequently, the field distribution includes both absolute amplitude and phase information (for more details on the phase alignment procedure, see Guderian and Düzel, 2005).

Next, sources of EEG oscillations within the spectro-temporal interval of interest were estimated by using a beamforming approach with multiple constraints. A beamformer is a spatial filtering method designed to obtain brain electrical activity from a specific location by attenuating contributions from other locations (e.g., Van Veen et al., 1997). However, beamformers have difficulty in separating EEG sources highly correlated in time. To overcome this drawback, we used the dual-core beamformer approach that allowed us to identify the main pairs of correlated sources that co-existed in each individual dataset (Diwakar et al., 2011). We performed 1000 searches for each dataset and selected all source pairs separated by more than 5 cm that were found in more than 1.5% of the searches. Afterwards, a multiple-constrained beamformer with correlated source suppression was applied to obtain the ultimate spatial filters from the time-of-

interest and baseline mean covariate matrices, respectively. This procedure generates pseudo-z maps for each trial, which are computed as time-of-interest/baseline power map ratios (Van Veen et al., 1997). This normalization step was applied to reduce the undesired contribution of noise power, which increases with depth and distance from sensors (Vrba and Robinson, 2001).

The cortical space was divided into a regular 5-mm voxel grid, and sensor outputs from unit dipoles in the three Cartesian directions were computed at each grid location. We used a realistic boundary-element model (Oostenveld et al., 2001) based on a standard template (Colin27 T1-weighten averaged MRI). This volume conduction model consists of 3 closed compartments with conductivities of 0.33, 0.0042 and 0.33 S/m corresponding to skin, skull, and brain, respectively. The regularization parameter was set at 0.001% of the largest eigenvalue of the covariance matrices.

After obtaining results derived from the corresponding statistical analyses, peak voxels and local maxima were selected to reconstruct the time course of EEG sources. We first obtained the optimal dipole orientation (Equation 8, Gross et al., 2001), then computed the power estimates using the voxel spatial filters and the mean covariate matrix of the time-of-interest described previously (Equations 4 and 5, Gross et al., 2001). Next, the optimal spatial filter was obtained by multiplying every optimal orientation value by its corresponding voxel spatial filters. The output of this computation was then multiplied by the filtered signal in the corresponding frequency bin of interest. Power decreases/increases relative to the baseline were computed in single-trial EEG source signals, as performed at the sensor level.

4. Statistical analysis of EEG oscillations at the source level

Firstly, the effect of age was removed from single-trial estimates at the source level using the general lineal model (Stolk et al., 2013). Secondly, we used a hierarchical statistical model to reduce the influence of inter-subject variability in oscillatory responses derived from across-subjects analyses (Holmes and Friston, 1998). Briefly, voxel-wise statistical analyses were computed for each participant on the original source activation maps obtained from all trials (or from congruent and incongruent trials, separately). Next, the corresponding contrast was applied at the group level. Individual analyses included comparisons with the baseline (one-sample *t*-tests), whereas at the

group level, we evaluated the main effect of group (HO vs. aMCI, aMCI-s vs. aMCI-c) and Spearman correlations (across-subjects and within each group, separately) with different memory indices (i.e., *associative d'* and *semantic d'*) on the *t*-maps or *r*-maps derived from the previous step. If these correlations reached statistical significance in at least one of the two groups, between-group analyses were performed using the Fisher method for independent samples applied to correlations. To this aim, *r*-maps were first transformed to *z*-score maps for each group and then the corresponding Fisher test was applied to obtain *z*-statistic values.

In order to avoid biasing the signal-to-noise ratio resulting from differences in the number of subjects per group, we first performed 1000 random draws of the data into two similar-sized groups, keeping the original assignment of the group fixed. Next, two-sample *t*-tests were applied on each draw, obtaining a distribution of 1000 *t*-values for each voxel. The statistical maps were obtained by computing the median *t*-value of each voxel distribution.

Family wise error (FEW) rates were controlled by applying non-parametric permutation tests combined with suprathreshold cluster analyses (Nichols and Holmes, 2002). Before cluster analysis, all *r*-, *t*- or *z*-statistic and mean activation maps were smoothed with an isotropic Gaussian kernel of 10 mm. The cluster was defined from the sum of the *t*-, *r*-, or *z*-values of contiguous voxels (cluster mass). After applying a primary threshold to voxels, *p*-values (α=0.05) were assessed by means of 1000 permutations using the Monte Carlo method implemented in the Fieldtrip toolbox. Coordinates of peak voxels and local maxima statistic values within each significant cluster were transformed into the Talairach space (Talairach and Tournoux, 1988) by using a nonlinear transformation. Cluster voxels were labeled according to the Brodmann area (BA) atlas included in the WFU Pick Atlas toolbox (Maldjian et al., 2003), using the same brain template employed for EEG source analyses. Finally, the signal of different local maximal was transformed into the time domain. The FWE rate across time was corrected with the maximum statistic approach (Nichols and Holmes, 2002).

5. Results at the sensor level

Fig. S1 shows the time-frequency representation (TFR) averaged across sensors and subjects for HO and aMCI individuals during the encoding (Fig. S1A) and memory task

(Fig. S1B). During these two phases, the two groups exhibited increased power in the low frequencies (i.e., delta and theta) with respect to the baseline during the entire interval of analysis following face presentation, as well as decreased power in alpha and beta bands from around 500 to 1000 ms from the face onset. However, PLS differences were restricted to the beta band. In particular, HO showed a significantly decrease of beta power (i.e., higher beta ERD) when compared with aMCI, both during the encoding (14-25 Hz; 0-1000 ms; $p < 0.04$) and subsequent memory task (13.5-18 Hz; 700-1000 ms; $p < 0.05$).

During encoding and within the beta band, HO could further be distinguished from both aMCI-c (13-16 Hz; 200-600 ms; $p < 0.05$) and aMCI-s individuals (13-17 Hz; 300-700 ms; $p < 0.03$). Similar results were obtained during the memory task, where HO showed higher beta ERD when compared with aMCI-c (13.5-18 Hz; 700-1000 ms; $p < 0.05$), although differences with aMCI-s did not achieve statistical significance.

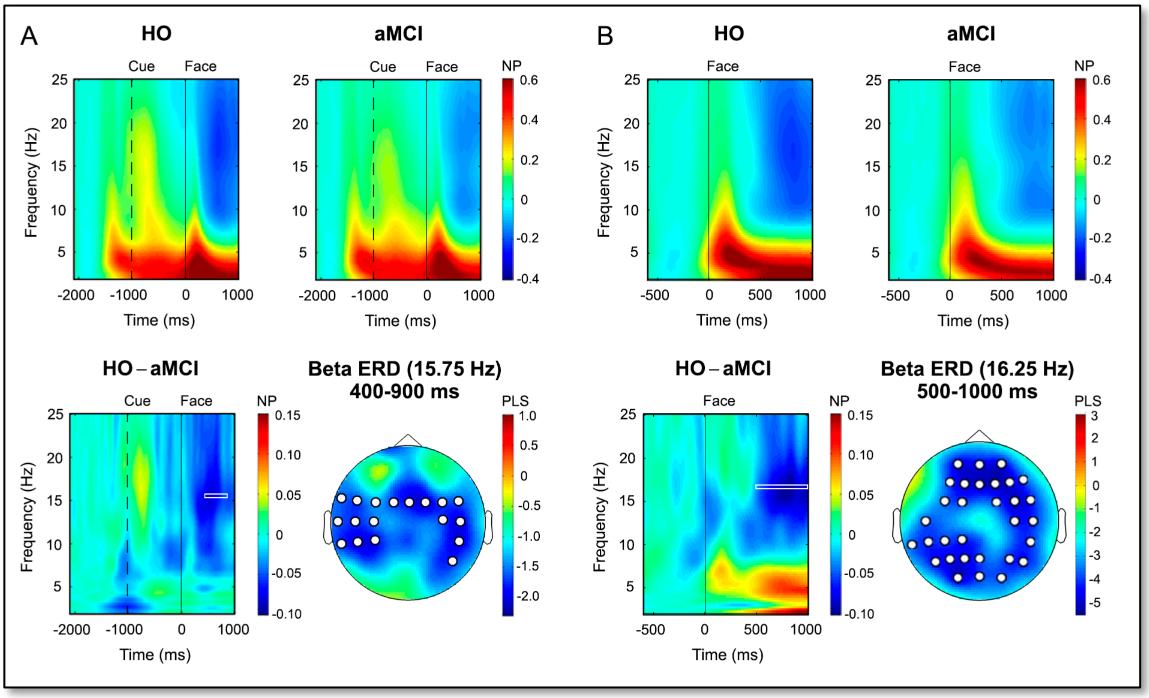

Figure S1. Group differences between HO and aMCI at the sensor level. Grand-average time-frequency representations (TFR) of normalized power (NP) for HO and aMCI (on the top) and difference between them (on the bottom) during both encoding (A) and retrieval (B). The small white frames in the difference TFR indicate the time-frequency windows used for source estimation of EEG beta power. Scalp distribution of values derived from PLS within these time-frequency windows are shown on the right. Open white circles over the scalp indicate sensors where differences in the NP between HO and aMCI reached statistical significance.

As illustrated in Fig. S2, aMCI-s showed higher alpha ERD than aMCI-c during the encoding (Fig. S2A) and memory task (Fig. S2B), for the whole 1000-ms poststimulus interval. However, the PLS only revealed significant differences between the two groups in the memory task (8-12 Hz; 0-1000 ms; $p < 0.04$).

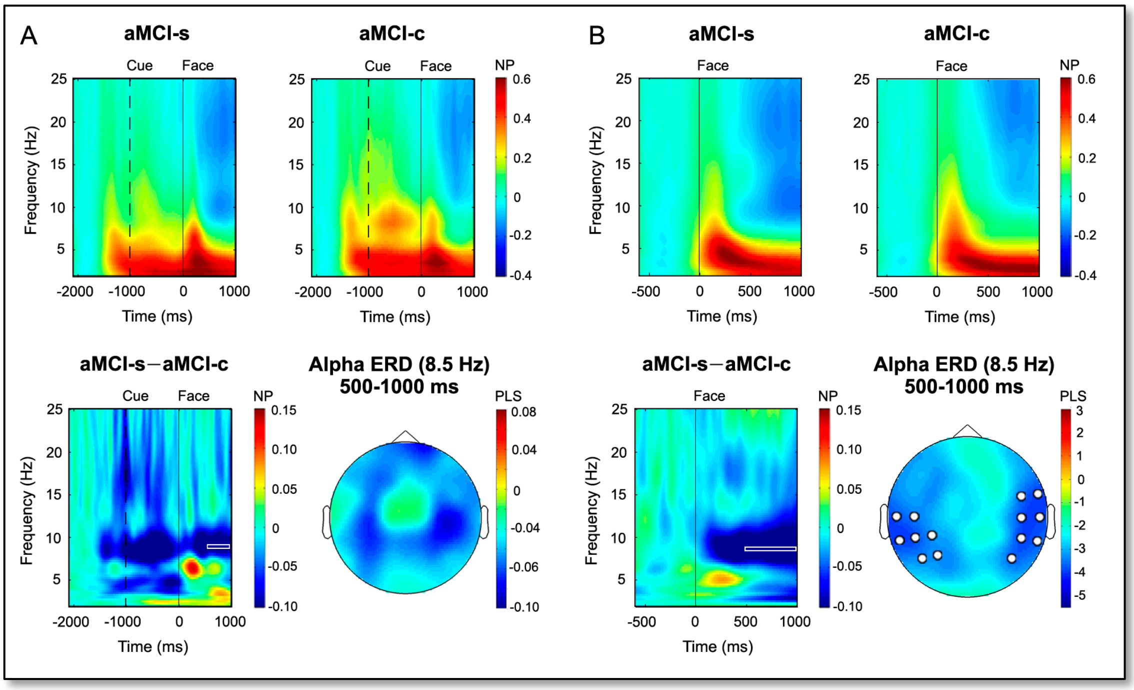

Figure S2. Group differences between aMCI-s and aMCI-c at the sensor level. Grand-average time-frequency representations (TFR) of normalized power (NP) for aMCI-s and aMCI-c (on the top) and difference between them (on the bottom) during both encoding (A) and retrieval (B). The small white frames in the difference TFR indicate the time-frequency windows used for source estimation of EEG alpha power. Scalp distribution of values derived from PLS within these time-frequency windows are shown on the right. Open white circles over the scalp indicate sensors where differences in the NP between aMCI-s and aMCI-c reached statistical significance. Note that these differences only achieved statistical significance during retrieval.

After averaging the EEG power over the sensors showing a significant decrease of beta power in the PLS, the maximum *t*-statistic after correcting for multiple comparisons across time was located at 15.75 Hz in the encoding task and at 16.25 Hz in the memory task at different time points depending on group comparisons. EEG sources for the former were estimated in the 400-900-ms interval, and for the latter in the 500-1000-ms window, given that the PLS within these time periods also revealed a significant decrease of power with respect to the baseline. For the alpha band, the maximum statistic after comparing the aMCI groups during the memory task was located at 8.5 Hz

in the 800 ms time point. Accordingly, the EEG sources of this activity were estimated within 500 and 1000 ms from face onset. As differences between aMCI-s and aMCI-c were also evident during encoding at 8.5 Hz within 700 and 1000 ms after applying the *t*-test ($p_{corrected} < 0.05$), and as the PLS showed a significant decrease with respect to the baseline from 500 up to 1000 ms after face presentation, we decided to further estimate EEG sources of this activity during encoding in the 500-1000-ms interval.

6. Differences in alpha/beta between HO and aMCI groups at the source level

During encoding, aMCI-c showed decreased alpha ERD with respect to HO ($p_{cluster} <$ 0.001) on a cortical widespread network comprising the fusiform, inferior temporal gyrus, temporoparietal junction and dorsolateral prefrontal cortex of the left hemisphere (Fig. S3A, Table S1). Differences with HO emerged in aMCI-c at around 200 ms after face presentation in the fusiform gyrus, to later spread to the temporal (at 312 ms), frontal (at 364 ms) and parietal lobe (at 468 ms).

Table S1. Cortical regions showing group differences in alpha ERD during the encoding and memory task.

**Contrast**<br>Cortical region	BA	*x*	*y*	*z*	*t*	Time (ms)	*T*	*p*
**ENCODING TASK**								
**HO > aMCI-c** $p_{\text{cluster}} < 0.001$								
L Precentral gyrus	6	-40	-3	28	-1.03	468-980	-3.39/-2.29	0.0003-0.03
L Middle frontal gyrus	6	-40	7	53	-1.01	364-980	-4.77/-3.07	0.0003-0.03
L Fusiform gyrus	19	-50	-73	-12	-0.90	208-980	-3.44/-2.25	0.002-0.04
L Inferior parietal lobe	40	-55	-28	33	-0.90	468-980	-3.33/-2.53	0.002-0.02
L Superior temporal gyrus	22	-65	-53	23	-0.89	312-980	-3.30/-2.21	0.002-0.04
L Inferior temporal gyrus	20	-65	-48	-22	-0.77	416-980	-3.11/-2.19	0.004-0.04
L Middle temporal gyrus	39	-45	-73	28	-0.76	520-980	-2.70/-2.29	0.01-0.03
**MEMORY TASK**								
**HO > aMCI-c** $p_{\text{cluster}} < 0.005$								
L Superior temporal gyrus	22	-65	-53	23	-0.97	460-980	-3.69/-2.59	0.0007-0.02
L Middle temporal gyrus	19	-55	-63	18	-0.94	408-980	-4.44/-2.51	0.0001-0.02

The direction of the contrast indicates that the first group showed higher low-alpha ERD (i.e., higher decrease of alpha power) than the second group. BA = Brodmann area; L = left; R = right; *t* = *t*-statistic at the source level; *T* = range of t-statistics in the time domain; *p* = range of *p* values in the time domain.

The aMCI-c group also showed decreased alpha ERD with respect to HO during retrieval, but differences were mostly limited to the left temporoparietal junction ($p_{cluster}$ < 0.005; Fig. S3B, Table S1). As in the encoding task, no differences were observed when the aMCI groups were compared.

Table S2. Cortical regions showing group differences in beta ERD during the encoding and memory task.

**Contrast** Cortical region	BA	*x*	*y*	*z*	*t*	Time (ms)	*T*	*p*
**ENCODING TASK**								
**HO > aMCI-s** $p_{\text{cluster}} < 0.03$								
R Superior temporal gyrus	38	60	12	-17	-2.27	364-980	-3.56/-2.34	0.0009-0.02
**HO > aMCI-c**								
$\boldsymbol{p_{\text{cluster}} < 0.003}$								
L Postcentral gyrus	2	-50	-13	33	-1.07	104-936	-5.05/-2.39	0.0001-0.02
L Inferior parietal lobe	40	-60	-38	43	-0.92	208-936	-4.31/-2.39	0.0001-0.03
L Middle frontal gyrus	6	-35	2	63	-0.92	312-980	-3.86/-2.33	0.004-0.03
L Superior frontal gyrus	6	-30	2	68	-0.90	312-980	-4.00/-2.27	0.0003-0.03
$\boldsymbol{p_{\text{cluster}} < 0.05}$								
L Inferior frontal gyrus	44	-60	22	8	-0.80	572-728	-2.44/-2.32	0.02-0.03
L Superior temporal gyrus	22	-55	12	-2	-0.76	416-676	-2.50/-2.26	0.02-0.03
**RETRIEVAL TASK**								
**HO > aMCI-c** $p_{\text{cluster}} < 0.02$								
R Postcentral gyrus	3	55	-8	48	-3.46	616-980	-3.82/-2.77	0.0004-0.01
R Supramarginal gyrus	40	65	-53	23	-3.38	668-876	-2.84/-2.51	0.006-0.02
R Inferior parietal lobe	40	60	-23	33	-3.34	616-980	-3.40/-2.45	0.002-0.02
R Superior temporal gyrus	22	65	-48	13	-3.31	512-928	-3.55/-2.34	0.001-0.03
R Middle occipital gyrus	18/19	10	-98	23	-3.24	512-980	-4.05/-2.67	0.0002-0.01
R Cuneus	18/19	15	-93	28	-3.18	564-928	-3.60/-2.24	0.0008-0.03
R Fusiform gyrus	37	40	-48	-12	-3.11	668-876	-2.69/-2.47	0.01-0.02
R Middle frontal gyrus	6/8	55	17	43	-2.84	616-980	-3.43/-2.46	0.002-0.02
L Lingual gyrus	17/18	-10	-103	-12	-2.75	720-928	-2.96/-2.42	0.005-0.02
L Superior frontal gyrus	6	-10	17	53	-2.57	408-980	-2.96/-2.09	0.005-0.02
R Parahippocampal gyrus	30	15	-33	-7	-2.53	720-876	-2.60/-2.48	0.01-0.02
L Anterior cingulate gyrus	24	-5	12	33	-2.48	512-980	-3.28/-2.37	0.002-0.02
R Precuneus	7	25	-58	43	-2.37	772-980	-2.80/-2.49	0.007-0.02
R Posterior cingulate gyrus	23	5	-28	28	-2.37	564-876	-2.71/-2.34	0.009-0.03

The direction of the contrast indicates that the first group showed higher low-beta ERD (i.e., higher decrease of beta power) than the second group. BA = Brodmann area; L = left; R = right; *t* = *t*-statistic at the source level; *T* = range of t-statistics in the time domain; *p* = range of *p* values in the time domain.

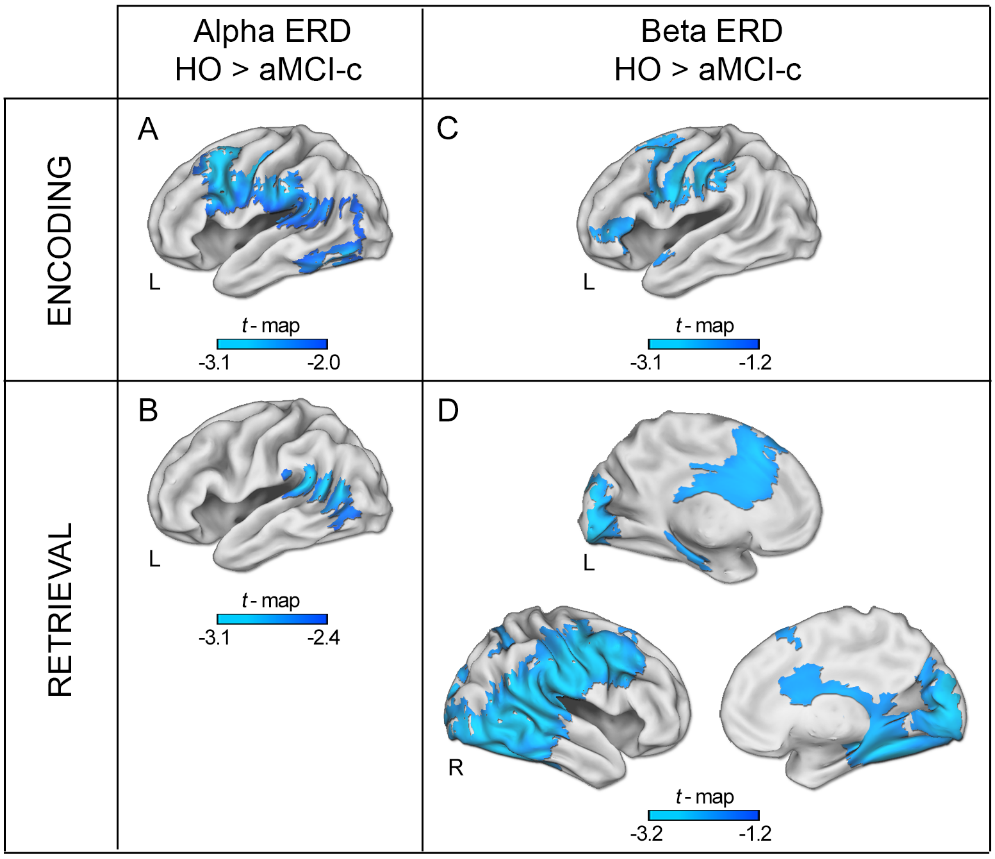

Figure S3. Group differences between HO and aMCI-c in alpha and beta band. Statistical nonparametric maps showing higher alpha ERD in HO than in aMCI-c during encoding (A) and retrieval (B), and higher beta ERD in HO than in aMCI-c during encoding (C) and retrieval (D). The blue gradient is mapped to statistical values (t-statistic). Peak voxels of significant clusters are listed in Table S1 and Table S2. L = left; R = right.

In the beta band, analyses further revealed significant differences between HO and the aMCI groups. During encoding, HO showed higher beta ERD in comparison with both the aMCI-s group in the right superior temporal gyrus ($p_{cluster} < 0.03$), and the aMCI-c group in the left inferior parietal lobe and left motor areas ($p_{cluster} < 0.003$) from very early in time (around 100-200 ms from face onset), and at about 600 ms in the left inferior frontal gyrus ($p_{cluster} < 0.05$; Fig. S3C, Table S2). However, during the memory task, HO showed lower beta power when compared to aMCI-c ($p_{cluster} < 0.02$), but not in comparison with aMCI-s. These differences were mostly evident from 400-500 ms onwards in the medial aspects of the left frontal, temporal and occipital lobe as well as in the lateral and medial aspects of a wide region in the right hemisphere mainly comprising the posterior part of frontal, parietal and temporal lobes (Fig. S3D, Table S2).

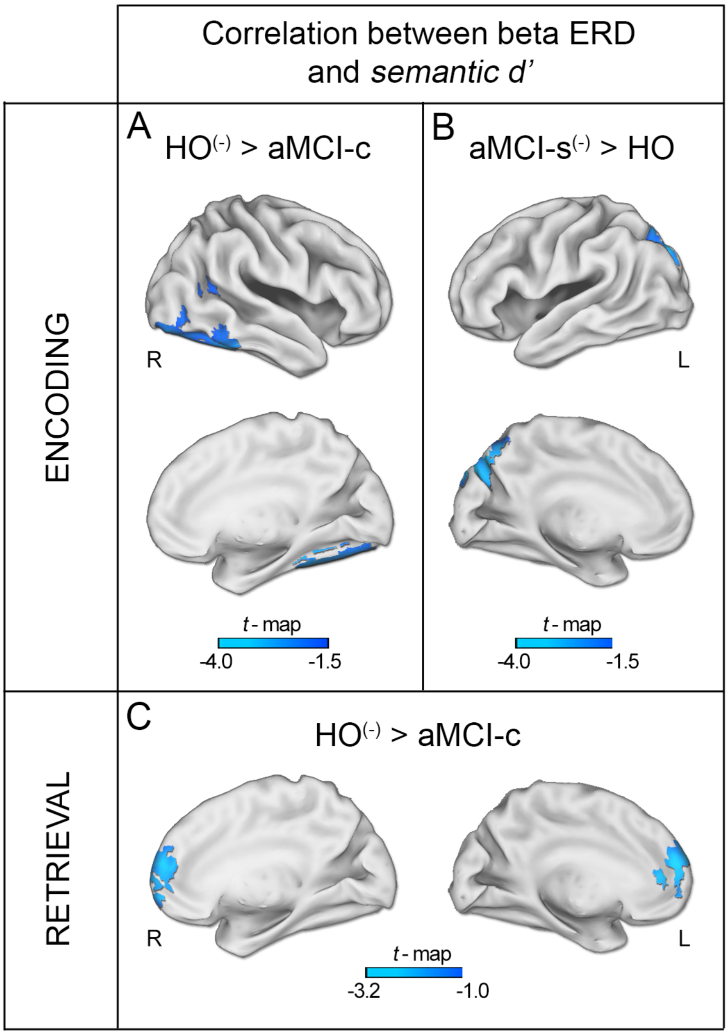

Figure S4. Relationship between beta ERD and the beneficial effect of semantic congruence on memory. Statistical nonparametric maps showing significant differences between HO and aMCI-c for the correlation between beta ERD and the *semantic d'* index during encoding (A) and retrieval (B). The blue gradient is mapped to statistical values (t-statistic). Peak voxels of significant clusters are listed in Table S3. L = left; R = right.

Table S3. Cortical regions showing group differences in correlations between beta ERD and *semantic d'* during the encoding and memory task.

**Contrast** Cortical region	BA	*x*	*y*	*z*	*t*	Time (ms)	*T*	*p*
**ENCODING TASK**								
**HO$^{(-)}$ > aMCI-c $p_{cluster} < 0.002$**								
R Parahippocampal gyrus	19	30	-48	-7	-2.82	676-980	-3.44/-2.49	0.007-0.05
R Inferior occipital gyrus	18	40	-88	-12	-2.65	0-980	-3.99/-2.57	0.001-0.02
R Middle temporal gyrus	19	40	-63	13	-2.07	0-980	-3.71/-1.43	0.007-0.04
**aMCI-s$^{(-)}$ > HO $p_{cluster} < 0.02$**								
L Cuneus	19	-15	-88	43	-3.27	572-832	-2.79/-2.44	0.007-0.02
L Precuneus	7	-10	-78	43	-3.23	572-936	-3.23/-2.58	0.007-0.03
**RETRIEVAL TASK**								
**HO$^{(-)}$ > aMCI-c$^{(+)}$ $p_{cluster} < 0.04$**								
L Superior frontal gyrus	8	-15	57	33	-3.28	512-824	-3.39/-2.59	0.006-0.04
L Medial frontal gyrus	9	0	47	18	-2.75	668-824	-2.74/-2.31	0.02-0.05

The sign (-) indicates the presence of a significant negative correlation in that particular group, meaning that the lower the beta power, the higher the benefit of semantic congruence in that particular group. The sign (+) indicates just the opposite. The direction of the contrast indicates that the first group showed a stronger correlation than the second group. BA = Brodmann area; L = left; R = right; *t* = *t*-statistic at the source level; *T* = range of t-statistics in the time domain; *p* = range of *p* values in the time domain.

HO showed a stronger negative correlation between the benefit of semantic congruence and differences in the power of beta ERD between SCF and SIF when compared to aMCI-c during encoding ($p_{cluster} < 0.002$) and retrieval ($p_{cluster} < 0.04$). During the encoding task, these differences appeared in the right fusiform and lingual gyrus from face onset until the end of the analysis time interval, whereas differences in the right parahippocampal cortex begun to be evident at around 700 ms until the end of the analysis interval (Figure S4A, Table S3). During the memory task, differences were only seen from 500 ms poststimulus onward in the medial prefrontal lobe (Figure S4C, Table S3). This correlation further distinguished the aMCI-s group from HO during encoding ($p_{cluster} < 0.02$). These results were limited to cuneus and precuneus in the left hemisphere ($p_{cluster} < 0.05$; Figure S4B, Table S3).

During retrieval, the aMCI-s group further showed a significantly stronger correlation between beta ERD and associative memory in a wide cortical network when compared with both HO ($0.005 < p_{cluster} < 0.02$) and aMCI-c ($p_{cluster} < 0.04$) individuals. The results are listed in Table S4.

Table S4. Cortical regions showing group differences in correlations between beta ERD and *associative d'* during the memory task.

Contrast<br>Cortical region	BA	*x*	*y*	*z*	*t*	Time (ms)	*T*	*p*
**aMCI-s(-) > HO**								
**$p_{cluster} < 0.005$**								
L Superior frontal gyrus	6	0	37	63	-3.56	668-1000	-3.29/-2.65	0.002-0.03
L Superior frontal gyrus	9/10	-15	57	28	-3.01	512-928	-3.57/-2.38	0.001-0.05
R Cingulate gyrus	31	20	-38	43	-2.81	304-928	-3.02/-2.43	0.02-0.05
L Middle frontal gyrus	46	-50	47	18	-2.79	616-1000	.21/-2.33	0.003-0.05
R Superior frontal gyrus	10	15	72	18	-2.76	460-1000	.92/-2.33	0.009-0.04
R Precuneus	7	10	-58	68	-2.73	304-876	.59/-2.29	0.02-0.04
L Cingulate gyrus	24	-10	17	23	-2.50	564-1000	.74/-2.39	0.006-0.03
**$p_{cluster} < 0.01$**								
R Lingual gyrus	17/18	10	-103	-12	-3.11	512-1000	-3.31/-2.61	0.008-0.04
R Cuneus	18	25	-103	3	-2.83	460-1000	-3.46/-2.57	0.003-0.05
R Middle occipital gyrus	18/19	35	-93	18	-2.33	616-772	-2.42/-2.26	0.01-0.03
**$p_{cluster} < 0.02$**								
R Middle frontal gyrus	10	50	52	-7	-3.26	460-1000	-3.34/-2.28	0.005-0.05
R Middle frontal gyrus	46	55	37	18	-3.06	668-1000	-3.13/-2.38	0.005-0.05
R Inferior frontal gyrus	45	45	27	8	-2.93	616-1000	-3.41/-2.59	0.007-0.03
R Insula	50	3	-2	13	-2.64	668-1000	-2.88/-2.54	0.002-0.006
R Superior temporal gyrus	22	60	-8	3	-2.37	772-1000	-2.67/-2.47	0.03-0.04
R Posterior cingulate	2	70	-18	13	-2.31	668-1000	.44/-2.37	0.008-0.009
R Supramarginal gyrus	40	50	-38	38	-2.12	824-928	.65/-2.47	0.03-0.04
R Middle frontal gyrus	10	50	52	-7	-3.26	460-1000	.34/-2.28	0.005-0.05

The sign (-) indicates the presence of a significant negative correlation in that particular group, which means that the lower the beta power, the higher the associative memory in that particular group. The direction of the contrast indicates that the first group showed a stronger correlation than the second group. BA = Brodmann area; L = left; R = right; t = t-statistic at the source level; T = range of t-statistics in the time domain; p = range of p values in the time domain.

7. Correlation between alpha power and associative memory in aMCI-s individuals.

Table S5. Cortical regions showing a significant correlation across aMCI-s individuals between *associative d'* and alpha ERD during the encoding and retrieval task.

**Contrast**<br>Cortical region	BA	*x*	*y*	*z*	*t*	Time (ms)	*T*	*p*
**ENCODING TASK**								
**aMCI-s**								
$p_{cluster} < 0.005$								
L Supramarginal gyrus	40	-60	-48	33	-0.65	468-988	-0.54/-0.81	10^{-4}-0.02
L Posterior cingulate	31	-20	-43	38	-0.63	676-988	-0.55/-0.6	0.009-0.01
L Inferior parietal lobe	40	-65	-38	28	-0.60	520-988	-0.52/-0.8	10^{-4}-0.02
L Parahippocampal gyrus	28	-20	-13	-17	-0.58	572-988	-0.55/-0.66	0.003-0.01
L Middle temporal gyrus	39	-40	-58	23	-0.53	676-936	-0.51/-0.57	0.01-0.03
**RETRIEVAL TASK**								
**aMCI-s**								
$p_{cluster} < 0.03$								
R Precuneus	7	20	-63	53	-0.73	512-980	-0.55/-0.81	10^{-4}-0.01
L Superior temporal gyrus	22	-65	-48	23	-0.69	408-980	-0.56/-0.80	10^{-4}-0.01
R Inferior temporal gyrus	37	60	-48	-22	-0.68	512-980	-0.53/-0.81	10^{-4}-0.02
R Fusiform gyrus	37	60	-63	-12	-0.67	460-980	-0.48/-0.81	10^{-4}-0.01
R Superior parietal lobule	7	25	-53	68	-0.63	408-980	-0.61/-0.83	0.001-0.02
L Inferior parietal lobule	40	-65	-33	23	-0.62	304-980	-0.54/-0.72	0.001
R Cuneus	19	25	-93	33	-0.61	564-980	-0.57/-0.78	0.001-0.01

BA = Brodmann area; L = left; R = right; *t* = *t*-statistic at the source level; *T* = range of t-statistics in the time domain; *p* = range of *p* values in the time domain.

References

Böhm P, Peña-Casanova J, Aguilar M, Hernández G, Sol JM, Blesa R. Clinical validity and utility of the interview for deterioration of daily living in dementia for Spanish-speaking communities NORMACODEM Group. Int Psychogeriatr 1998; 10:261-70.

Diwakar M, Huang MX, Srinivasan R, Harrington DL, Robb A, Angeles A, Muzzatti L, Pakdaman R, Song T, Theilmann RJ, Lee RR. Dual-Core Beamformer for obtaining highly correlated neuronal networks in MEG. Neuroimage 2011; 54:253-63.

Fazekas F, Chawluk JB, Alavi A, Hurtig HI., Zimmerman RA. MR signal abnormalities at 1.5 T in Alzheimer's dementia and normal aging. Am J Neuroradiol 1987; 8:421-6.

Gross J, Kujala J, Hämäläinen M, Timmermann L, Schnitzler A, Salmelin R. Dynamic imaging of coherent sources: Studying neural interactions in the human brain. Proc Natl Acad Sci USA 2001; 98:694-9.

Guderian S, Düzel E. Induced theta oscillations mediate large- scale synchrony with mediotemporal areas during recollection in humans. Hippocampus 2005; 15:901-12.

Holmes AP, Friston KJ. Generalisability, random effects and population inference. Neuroimage 1998; 7:S754.

Hughes CP, Berg L, Danziger WL, Coben LA, Martin RL. A new clinical scale for the staging of dementia. Brit J Psychiat 1982; 140:566-72.

Lobo A, Escobar V, Ezquerra J, Seva Díaz A. "El Mini-Examen Cognoscitivo"(Un test sencillo, práctico, para detectar alteraciones intelectuales en pacientes psiquiátricos). Rev Psiquiat Psicol Med 1980; 14:39-57.

Maldjian JA, Laurienti PJ, Kraft RA, Burdette JH. An automated method for neuroanatomic and cytoarchitectonic atlas-based interrogation of fMRI data sets. Neuroimage 2003; 19:1233-9.

Maris E. Randomization tests for ERP topographies and whole spatiotemporal data matrices. Psychophysiology 2004; 41:142-51.

Nichols TE, Holmes AP. Nonparametric permutation tests for functional neuroimaging: A primer with examples. Hum Brain Mapp 2002; 15:1-25.

Oostenveld R, Praamstra P, Stegeman DF, van Oosterom A. Overlap of attention and movement-related activity in lateralized event-related brain potentials. Clin Neurophysiol 2001; 112:477-84.

Stolk A, Todorovic A, Schoffelen JM, Oostenveld R. Online and offline tools for head movement compensation in MEG. Neuroimage 2013; 68:39-48.

Talairach J, Tournoux P. Co-planar stereotaxic atlas of the human brain. New York: Thieme Medical Publishers. 1988.

Van Veen BD, Van Drongelen W, Yuchtman M, Suzuki A. Localization of brain electrical activity via linearly constrained min- imum variance spatial filtering. IEEE Trans Biomed Eng 1997; 44:867-80.

Vrba J, Robinson SE. Signal processing in magnetoencephalography. Methods 2011; 25:249-71.

Wechsler, D. Wechsler Memory Scale—Third Edition Manual. San Antonio, TX: The Psychological Corporation. 1997.

Yesavage JA, BrinK TL, Rose TL, Lum O. Development and validation of a geriatric depression scale: a preliminary report. J Psychiatr Res 1983; 17:37-49.

Petersen RC, Smith GE, Waring SC, Ivnik RJ, Tangalos EG, Kokmen E. Mild cognitive impairment: clinical characterization and outcome. Arch Neurol 1999; 56:303-8.

Winblad B, Palmer K, Kivipelto M, Jelic V, Fratiglioni L, Wahlund LO, Nordberg A, Bäckman L, Albert M, Almkvist O, Arai H, Basun H, Blennow K, de Leon M, DeCarli C, Erkinjuntti T, Giacobini E, Graff C, Hardy J, Jack C, Jorm A, Ritchie K, van Duijn C, Visser P, Petersen RC.Mild cognitive impairment - beyond controversies, towards a consensus. J Intern Med 2004; 256:240-6.
